# Phase II trial of individualized/dynamic cisplatin regimens for definitive concurrent chemoradiation therapy in patients with head and neck squamous cell carcinoma

**DOI:** 10.1002/cam4.3529

**Published:** 2020-10-19

**Authors:** Dongbin Ahn, Gil Joon Lee, Jin Ho Sohn, Jeong Eun Lee

**Affiliations:** ^1^ Department of Otolaryngology‐Head and Neck Surgery School of Medicine Kyungpook National University Daegu Korea; ^2^ Department of Radiation Oncology School of Medicine Kyungpook National University Daegu Korea

**Keywords:** chemoradiotherapy, cisplatin, head and neck squamous cell carcinoma, performance status, protocol, toxicity

## Abstract

The current standard cisplatin regimen for concurrent chemoradiation therapy (CCRT) involves generalized static administration of cisplatin without considering patient characteristics and patient/tumor responses during treatment. We aimed to evaluate the oncological feasibility of individualized/dynamic cisplatin regimens for definitive CCRT in patients with head and neck squamous cell carcinoma (HNSCC). This prospective, single‐center study enrolled patients with biopsy‐confirmed HNSCC for whom CCRT was indicated as the primary treatment. Concurrent with radiation therapy (RT), patients received individualized and dynamically modified cisplatin chemotherapy based on patient characteristics, such as age and Eastern Cooperative Oncology Group performance status (PS), and patient/tumor treatment responses. The primary endpoints of the study were grade ≥3 toxicity and progression‐free survival (PFS). The study enrolled 150 patients; 146 (97.3%) received ≥2 cycles of cisplatin in addition to scheduled RT. Incidence of any grade 3‐4 toxicities was 40.7% (61/150). During the 40.1 ± 25.1‐month follow‐up period, the 2‐year locoregional control, distant control, PFS, disease‐specific survival, and overall survival were 81.7%, 89.2%, 73.0%, 89.2%, and 86.1%, respectively. The treatment compliance and grade ≥3 toxicities did not differ between patients aged <70 years and ≥70 years, or those with PS 0 and PS 1‐2, respectively. CCRT using individualized, dynamic cisplatin regimens based on patient age, PS, and patient/tumor responses during treatment was oncologically safe and effective for treating patients with HNSCC, including those aged ≥70 years and with PS 1‐2.

## Graphical abstract

Concurrent chemoradiation therapy using individualized/dynamic cisplatin regimen, based on patient age, performance status (PS), and patient/tumor responses during treatment, results in favorable oncological outcomes with considerably low toxicity in patients with head and neck squamous cell carcinoma. These cisplatin regimens present useful alternatives to the currently used cisplatin regimen, particularly in elderly patients with declining PS.

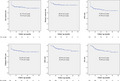

## 1 INTRODUCTION

Concurrent chemoradiation therapy (CCRT) with three cycles of tri‐weekly high‐dose (100 mg/m^2^) cisplatin is the current standard for the definitive treatment of advanced head and neck squamous cell carcinoma (HNSCC) and the preferred treatment choice in major international practice guidelines.^1^, ^2^, ^3^, ^4^, ^5^, ^6^, ^7^ Notwithstanding the survival benefits that can be achieved with CCRT over radiation therapy (RT) alone, toxicities related to high‐dose cisplatin present considerable obstacles to the completion of the treatment regimen.^1^, ^2^, ^3^, ^4^ According to three large randomized trials on CCRT with a tri‐weekly high‐dose cisplatin regimen, overall grade 3‐4 acute toxicities occurred in 77‐85% of patients, and treatment‐related death occurred in up to 5.3% of the cohort.^1^, ^3^, ^4^ In addition, there are concerns that treatment‐related toxicities could be more frequent and critical in elderly patients with diminished performance status (PS) and could possibly lead to lower treatment compliance and ultimately treatment failure.^6^, ^8^, ^9^

To decrease the therapeutic burden of high‐dose cisplatin regimens, there have been growing efforts to minimize acute toxicity without compromising the anticancer effect by tweaking several parameters of the cisplatin, such as peak dose, dose intensity, cumulative dose, and timing of delivery.^10^, ^11^, ^12^, ^13^ Recently, weekly low‐dose cisplatin regimens have gradually gained clinical acceptance, replacing the standard tri‐weekly schedule at some institutions.^10^ However, this regimen has little support from large comparative phase III trials, and several systematic reviews have failed to demonstrate the true benefits of weekly low‐dose regimens in survival outcomes as well as toxicity evaluation.^6^, ^10^, ^11^, ^14^, ^15^ Although a recent phase III trial showed significantly lower incidence of grade ≥3 toxicity in the weekly low‐dose cisplatin arm (71.6%) than in the tri‐weekly high‐dose cisplatin arm (84.6%), the incidence was still high and ultimately unsatisfactory.^16^

Current cisplatin regimens, regardless of weekly low‐dose or tri‐weekly high‐dose regimens, are generalized and static regimens that infuse a fixed standard dose of cisplatin in every chemotherapy cycle without considering patient characteristics and patient/tumor responses during treatment. However, given that drug susceptibility can depend on the patient and changes in patient/tumor status as CCRT progresses, such conventional regimens would not be a reasonable approach to minimizing toxicity or providing bespoke cisplatin doses for individual patient requirements.^17^ Instead, a more tailored standard dose determination along with dynamic dose modifications during treatment, would be a more reasonable approach to reduce drug toxicity without compromising the anticancer effect of cisplatin. In fact, although cisplatin dose modification during CCRT is a common practice in the clinical setting, its indication, protocols, and results have not been evaluated prospectively; consequently, there are no established recommendations or guidelines concerning this practice.^6^, ^8^, ^18^ Therefore, this phase II study evaluated the oncological feasibility of individualized/dynamic cisplatin regimens for definitive CCRT in patients with HNSCC.

## 2 MATERIALS AND METHODS

### 2.1 Patients

This study was designed as a single‐center, prospective phase II trial. The institutional review board of our institution approved the study protocol, and written informed consent was obtained from all patients. The study followed the ethical principles of the Declaration of Helsinki.

Patients were eligible for the trial if they had biopsy‐confirmed HNSCC in which CCRT was indicated as a primary treatment with curative intent according to the National Comprehensive Cancer Network Clinical Practice Guidelines in Oncology.^7^ Although this eligibility generally involved stage III and IV disease without distant metastasis based on the eighth edition of the American Joint Committee on Cancer (AJCC) staging system, some patients with locoregionally advanced disease (T3 or N1‐2) also classified as stage I or II by the eighth AJCC staging system were eligible, particularly in patients with human papilloma virus‐positive oropharyngeal and nasopharyngeal SCC. Patients who had head and neck malignancies other than SCC, who required CCRT as an adjuvant or palliative treatment, in whom chemotherapy agents other than cisplatin were indicated, and who had a history of previous head and neck malignancy were not eligible for this trial. Other eligibility criteria included an Eastern Cooperative Oncology Group (ECOG) PS of 0‐2, life expectancy of >12 weeks, adequate hematological condition (i.e., white blood cell [WBC] count ≥4000/µl, hemoglobin ≥10 g/dl, and platelet count ≥100,000/µl), glomerular filtration rate ≥60 ml/min, and no current or recent history of infections.

### 2.2 CCRT using individualized/dynamic cisplatin regimens

We used tri‐weekly cisplatin regimens in this trial. The cisplatin was scheduled to be administered as a 1‐h intravenous infusion on days 1, 22, and 43 of RT. Although the current standard dose for the tri‐weekly cisplatin regimen is 100 mg/m^2^, we tailored the dose according to patient age and ECOG PS and modified the dose for every chemotherapy cycle based on patient/tumor responses during the CCRT.

Table 1 shows the individualized standard dose of cisplatin, which ranged from 100 to 60 mg/m^2^. With this standard dose as an initial dose for each patient, cisplatin doses during the second and third chemotherapy cycles were dynamically modified based on the toxicity grade, change in the patient PS, and tumor response to the treatment (Table 2). For example, if PS was unchanged and/or grade 0‐1 toxicity was identified after the first cisplatin cycle, the second cisplatin dose was not modified. If PS increased by one grade and/or grade 1‐2 toxicity was observed, the cisplatin dose was reduced by 20‐25%. If PS increased by two grades and/or grade 2‐3 toxicity was observed, chemotherapy was postponed, and patient condition was reevaluated 1 week later. In select patients with an excellent treatment response during CCRT, the cisplatin dose was also modified. If gross tumor volume was reduced by ≥50% (partial response, PR) after the first cisplatin cycle, the second cisplatin dose was reduced from 20 to 25% of the first dose. If the tumor disappeared (complete response, CR) after the second cisplatin cycle, the third cisplatin dose was reduced by 20‐25% from the second dose. However, no dose was reduced to <60 mg/m^2^ in any patients; thus, at least 60 mg/m^2^ cisplatin was administered in each cycle.

**TABLE 1:** Individualized standard cisplatin doses based on the age and Eastern Cooperative Oncology Group performance status (PS)

	PS 0 (mg/m^2^)	PS 1 (mg/m^2^)	PS 2 (mg/m^2^)
<70 years	100	80	80
70‐79 years	80	80	60
≥80 years	80	60	60

**TABLE 2:** Protocol of dynamic dose modification during treatment

Assessment parameters			Decision of modification
PS	Toxicity	Tumor response	
No change	Grade 0‐1	—	None
+1 grade	Grade 1‐2	≥50% reduction of initial tumor volume prior to second cycle No visible tumor prior to third cycle	20‐25% reduction^a^
+2 grade	Grade 2‐3	—	1‐week postponement
≥3	Persistent grade 3‐4	—	Consider withdrawal

Abbreviations: PS, Eastern Cooperative Oncology Groupperformance status.

^a^ Dose reduction to <60 mg/m^2^ was not permitted.

For RT, an intensity‐modulated radiotherapy technique was used with 2.0 Gy/day administered for 5 days a week, at a total dose of 70 Gy in 35 fractions to the primary site and neck metastasis. Elective neck irradiation up to 45‐50 Gy was given to tumor‐free areas when indicated.

### 2.3 Assessment of treatment response and toxicity during CCRT

Patients were regularly followed up weekly after treatment initiation to evaluate their response and toxicity during CCRT. Response to the treatment regimen was evaluated based on office‐based modalities prior to each chemotherapy cycle. For the primary tumor, treatment response during CCRT was primarily evaluated via laryngoscopic/endoscopic examination. Although these examinations per se did not involve three‐dimensional (3D) images or allow direct measurement of tumor diameter in the exact measurement unit (cm), relative tumor size and volume could be estimated using the picture archiving and communication system, which enabled comparison of relative tumor size and volume between initial and follow‐up periods during treatment. For the neck metastasis, ultrasonography examination was used to evaluate treatment response by measuring the 3D diameter (cm) of the tumor and calculating tumor volume with the following equation: *V* (cm^3^) = *πabc*/6, where *V* is volume; *a*, the largest diameter (cm); and *b* (cm) and *c* (cm), the other two perpendicular diameters. This allowed comparison of tumor volume between initial and follow‐up periods during treatment.

Toxicity and adverse effects were monitored with medical history and abovementioned office‐based modalities, as well as laboratory studies including complete blood counts and serum biochemistry tests. Evaluation of toxicity was based on the fifth version of the Common Toxicity Criteria for Adverse Events.

### 2.4 Assessment of treatment response after CCRT

Conventional definitions were used to describe the treatment responses. The response was assessed 8‐12 weeks after completion of the CCRT by physical examination, laryngoscopic/endoscopic examination, computed tomography (CT), and positron emission tomography‐CT. If any suspicious remnant lesion was identified, a biopsy was performed to confirm disease status. Salvage surgery was recommended for patients who failed to achieve CR after completing CCRT or who experienced recurrence during follow‐up after achieving CR as long as curative surgical management of the disease was still possible.

### 2.5 Study endpoints and statistical analysis

The major endpoint of the study to demonstrate weather an individualized/dynamic cisplatin regimen resulted in acceptable oncological outcomes minimizing severe toxicities was grade 3‐4 toxicity and progression‐free survival (PFS). Secondary endpoints included CR rate, locoregional (LR) control, distant control, ultimate PFS (PFS after completion of overall treatment, including primary CCRT and salvage surgery), disease‐specific survival (DSS), and overall survival (OS). Survival data were analyzed using the Kaplan‐Meier method and the significance of difference was tested by log‐rank tests between subgroups. Survival was calculated from the date of completion of CCRT.

As a subgroup analysis, the results of CCRT, including treatment compliance, toxicity, and oncological outcomes, were evaluated in patients aged ≥70 years and with PS 1‐2 who were primary candidates for receiving a tailored cisplatin dose in this study. To evaluate the impact of the major clinicopathological characteristics on treatment failure, a Cox proportional hazards regression model was used, and the results are presented as hazard ratios (HRs) with 95% confidence intervals (CIs) and *p*‐values.

SPSS for Windows (version 18.0; SPSS Inc.) was used to analyze the data. To evaluate the results, *p*‐values were two‐sided throughout, and statistical significance was defined as *p* < 0.05.

## 3 RESULTS

### 3.1 Baseline patient characteristics

From January 2012 to December 2019, 150 patients participated in this study (Table 3). Of the total 150 patients, 127 patients (84.7%) were male and 38 patients (25.3%) were ≥70 years old. The primary tumor sites were the larynx, oropharynx, nasopharynx, and hypopharynx in 52 (34.7%), 38 (25.3%), 24 (16.0%), and 21 (14.0%) patients, respectively. According to the eighth AJCC staging system, 105 patients (70.0%) had stage III‐IV disease, and 45 patients had stage I‐II disease with T3 or N1‐2.

**TABLE 3:** Baseline patient characteristics

	Patients (N = 150)
Sex	
Male	127 (84.7%)
Female	23 (15.3%)
Age	
Mean ±standard deviation (years)	62.2 ± 11.0
<70 years	112 (74.7%)
70 years	38 (25.3%)
Performance status	
0	78 (52.0%)
1	41 (27.3%)
2	31 (20.7%)
Primary sites	
Nasal cavity/paranasal sinus	4 (2.7%)
Nasopharynx	24 (16.0%)
Oral cavity	6 (4.0%)
Oropharynx	38 (25.3%)
Larynx	52 (34.7%)
Hypopharynx	21 (14.0%)
Unknown	5 (3.3%)
T stage	
0	5 (3.3%)
1	24 (16.0%)
2	42 (28.0%)
3	47 (31.3%)
4	32 (21.3%)
N stage	
0	53 (35.3%)
1	33 (22.0%)
2	47 (31.3%)
3	17 (11.3%)
Overall stage	
1	18 (12.0%)
2	27 (18.0%)
3	38 (25.3%)
4	67 (44.7%)

Stage was classified according to eighth American Joint Committee on Cancer staging system.

### 3.2 Compliance and toxicity

One hundred forty‐six patients (97.3%) received two or more cycles of cisplatin with planned RT and 119 patients (79.3%) received planned cisplatin cycles. The maximum cumulative dose of cisplatin (300 mg/m^2^) was administered in only seven patients (4.7%), whereas cisplatin doses were reduced at any cisplatin cycle in the remaining 143 patients (95.3%). In 25 patients (16.7%), the cisplatin dose was reduced based on tumor response during CCRT, regardless of toxicity. The mean cumulative cisplatin dose was 212.3 ± 54.7 mg/m^2^. All patients received their planed radiation dose.

The most common toxicity was anemia, with 94.0% overall incidence, followed by nausea (91.3%) and mucositis (91.3%). Most common grade 3‐4 toxicities were mucositis (26.7%), followed by leukopenia (16.0%) and vomiting (9.3%). Incidence of any grade 3‐4 toxicities was 40.7% (61/150). Scheduled chemotherapy was delayed by at least ≥1 week in 41 patients (27.3%), and hospitalization for the management of toxicities was required in 31 patients (20.7%). Transient and permanent tube feeding was required in six patients (4.0%) and one patient (0.7%), respectively. No treatment‐related deaths occurred during the trial (Table 4).

**TABLE 4:** Toxicity

	Patients (N = 150)
Hematologic (overall/grade 3‐4)	
Anemia	141 (94.0%) / 9 (6.0%)
Leukopenia	121 (80.7%) / 24 (16.0%)
Thrombocytopenia	40 (26.7%) / 2 (1.3%)
Febrile neutropenia	1 (0.7%) / 1 (0.7%)
Non‐hematologic (overall/grade 3‐4)	
Nausea	137 (91.3%) / 11 (7.3%)
Vomiting	111 (74.0%) / 14 (9.3%)
Mucositis	137 (91.3%) / 40 (26.7%)
Radiation dermatitis	105 (70.0%) / 4 (2.7%)
Nephrotoxicity	8 (5.3%) / 0 (0.0%)
Infection	8 (5.3%) / 3 (2.0%)
Any grade 3‐4 toxicity	61 (40.7%)
Chemotherapy delay due to toxicity	41 (27.3%)
Hospitalization due to toxicity	30 (20.0%)
Tube feeding (transient/permanent)	6 (4.0%) / 1 (0.7%)
Treatment‐related death	0 (0.0%)

### 3.3 Oncological outcomes

The disease assessment after CCRT indicated that CR was achieved in 123 patients (82.0%) and not in 16 (10.7%), 11 (7.3%), and 9 (6.0%) patients in the local, regional, and distant sites, respectively.

After follow‐up of 40.1 ± 25.1 months, LR failure occurred in 30 patients (20.0%), including 19 patients with non‐CR and 11 patients with recurrence. The 2‐ and 5‐year LR control rates were 81.7% and 76.7%, respectively. Distant failure occurred in 16 patients (10.7%), and the 2‐ and 5‐year distant control rates were 89.2% and 87.8%, respectively. Overall treatment failure occurred in 42 patients (28.0%), and the 2‐ and 5‐year PFS were 73.0% and 67.8%, respectively. Among the 42 patients with treatment failure in definitive CCRT, 12 patients were salvaged with surgery with/without adjuvant treatment; thus, ultimate treatment failure occurred in 30 patients (20.0%). The 2‐ and 5‐year ultimate PFS were 81.4% and 77.5%, respectively.

**FIGURE 1:**
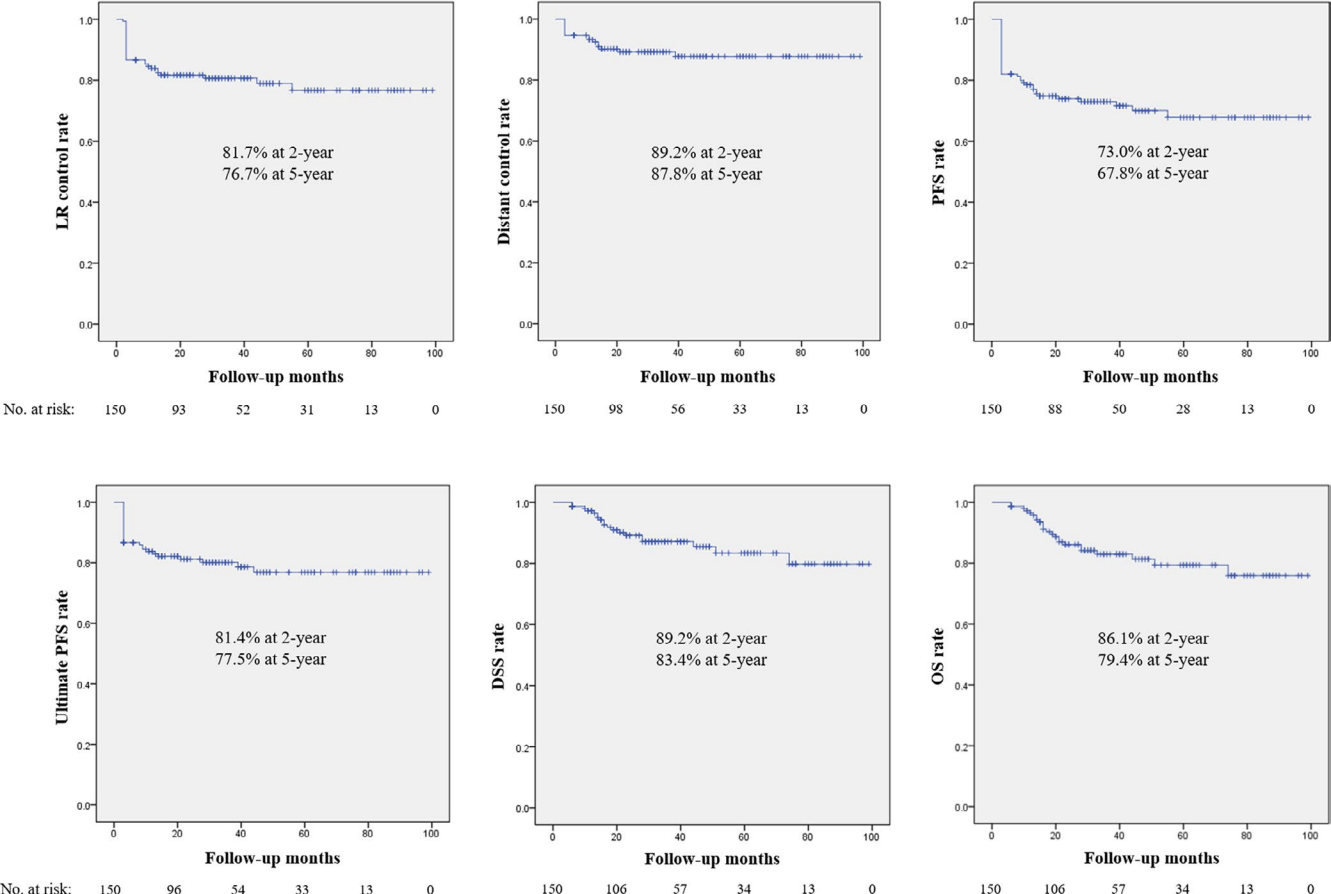
Kaplan‐Meier curves for oncological outcomes. DSS, disease‐specific survival; LR, locoregional; OS, overall survival; PFS, progression‐free survival

Twenty‐four patients (16.0%) died. Among these patients, non‐disease‐related deaths occurred in four patients (pneumonia in two patients and lung cancer in two patients). The 2‐ and 5‐year DSS were 89.2% and 83.4%, respectively; the 2‐ and 5‐year OS were 86.1% and 79.4%, respectively (Figure 1).

### 3.4 Results of CCRT in patients aged ≥70 years and with initial ECOG PS 1‐2

**FIGURE 2:**
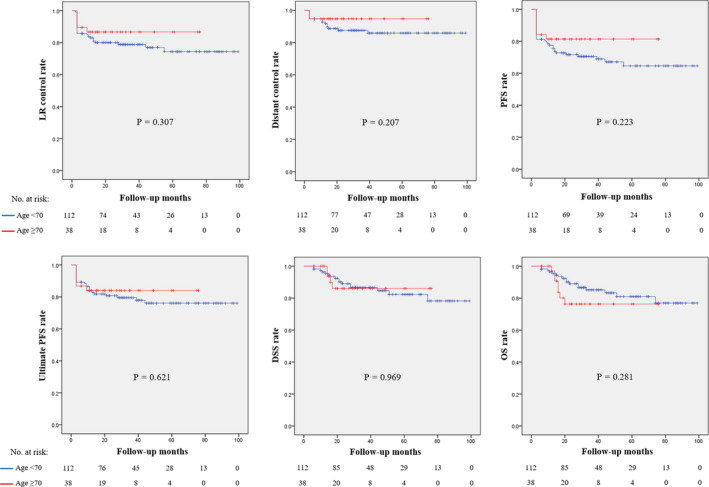
Kaplan‐Meier curves for comparing oncological outcomes between patients aged <70 and ≥70. DSS, disease‐specific survival; LR, locoregional; OS, overall survival; PFS, progression‐free survival

Table 5 summarizes the treatment compliance and toxicities of CCRT according to patient age and initial ECOG PS. In comparison with patients aged <70 years and ≥70 years, patients receiving ≥2 cycles of cisplatin, patients receiving all planned cycles of cisplatin, incidence of grade 3 toxicities, and incidence of hospitalization for toxicity management were not significantly different. The incidence of chemotherapy delay due to toxicity was considerably lower in patients aged ≥70 years compared with patients aged <70 years (15.8% vs. 31.3%, *p* = 0.065). A cumulative cisplatin dose was significantly lower in patients aged ≥70 years compared with patients aged <70 years (172.9 mg/m^2^ vs. 225.7 mg/m^2^, *p* < 0.001). Oncological outcomes including LR control, distant control, PFS, ultimate PFS, DSS, and OSS were not significantly different between patients aged <70 years and ≥70 years (Figure 2).

**TABLE 5:** Summary of treatment compliance and toxicity in patients aged ≥70 years and with PS 1‐2

	Age <70 (n = 112)	Age ≥70 (n = 38)	*p*‐value	PS 0 (n = 78)	PS 1‐2 (n = 72)	*p*‐value
Patients receving ≥2 cisplatin cycle	110 (98.2%)	36 (94.7%)	0.266	77 (98.7%)	69 (95.8%)	0.351
Patients receving all planned cisplatin cycle	90 (80.4%)	27 (71.1%)	0.232	58 (74.6%)	50 (69.4%)	0.503
Cumulative cisplatin dose (mg/m^2^)	225.7 ± 50.8	172.9 ± 46.6	<0.001	231.2 ± 43.9	191.8 ± 58.1	<0.001
Any grade 3‐4 toxicity	53 (47.3%)	15 (39.5%)	0.401	36 (46.2%)	19 (46.3%)	0.834
Chemotherapy delay due to toxicity	35 (31.3%)	6 (15.8%)	0.065	20 (25.6%)	16 (39.0%)	0.628
Hospitalization due to toxicity	21 (18.8%)	9 (23.7%)	0.551	13 (16.7%)	11 (26.8%)	0.288

**FIGURE 3:**
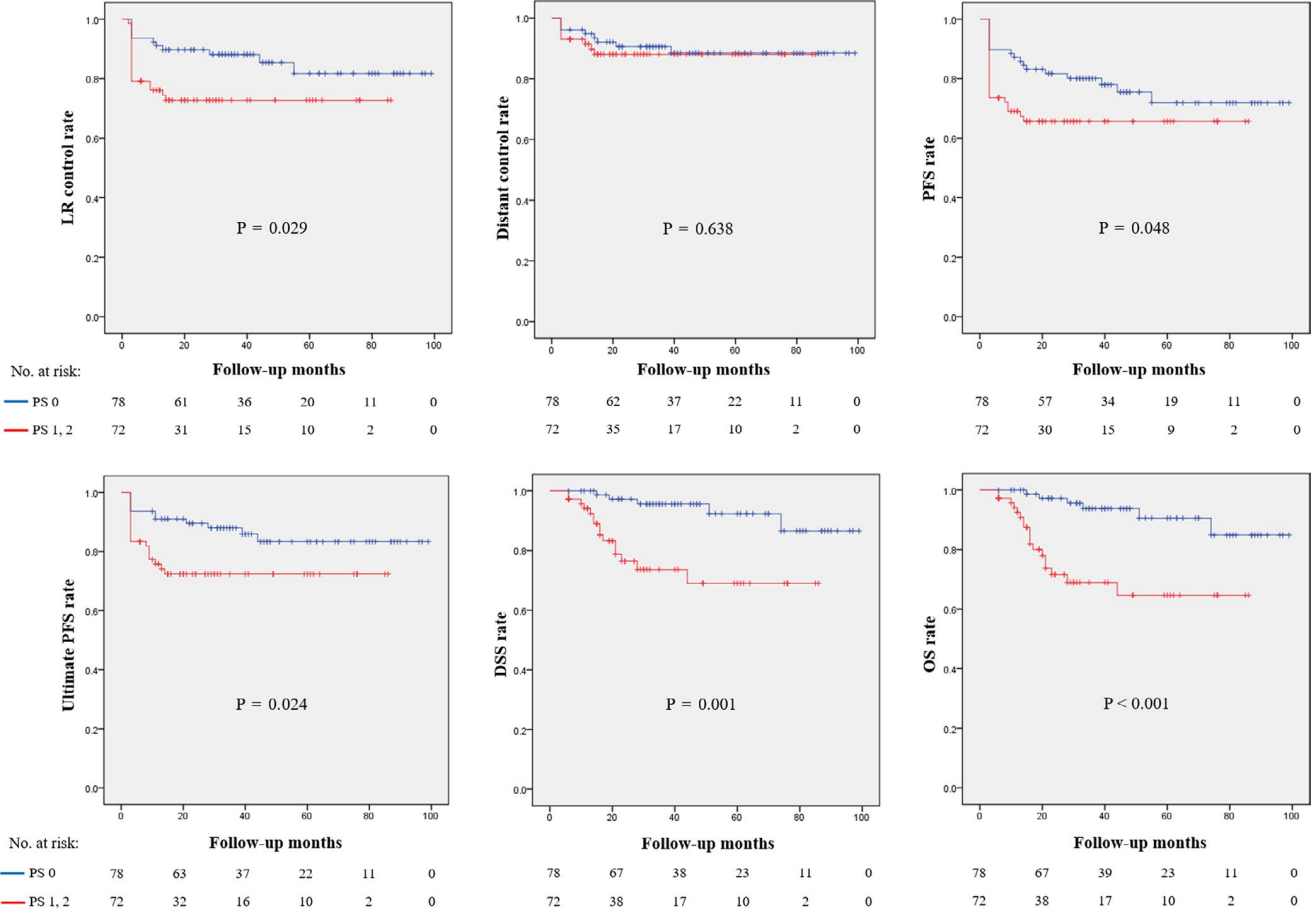
Kaplan‐Meier curves for comparing oncological outcomes between patients with PS 0 and PS 1‐2. DSS, disease‐specific survival; LR, locoregional; OS, overall survival; PFS, progression‐free survival; PS, performance status

In comparison with patients with PS 0 and PS 1‐2, patients receiving ≥2 cycle of cisplatin, patients receiving all planned cycles of cisplatin, incidence of grade 3 toxicities, incidence of chemotherapy delay due to toxicity, and incidence of hospitalization for toxicity management were not significantly different. A cumulative cisplatin dose was significantly lower in patients with PS 1‐2 compared with patients with PS 0 (191.8 mg/m^2^ vs. 231.2 mg/m^2^, *p* < 0.001). The oncological outcomes, including LR control, PFS, ultimate PFS, DSS, and OS, were significantly better in patients with PS 0 than PS 1‐2. Distant control was not different between patients with PS 0 and PS 1‐2 (Figure 3).

### 3.5 Factors associated with treatment failure

In the univariate analysis, only stage III‐IV disease was significantly associated with increased risk of treatment failure (HR, 4.377; 95% CI, 1.560‐12.284; *p* = 0.005). Although PS 1‐2 was also associated with increased risk of treatment failure, the statistical significance was not verified (HR, 1.713; 95% CI, 0.925‐3.171; *p* = 0.087). In the multivariate analysis, PS 1‐2 (HR, 2.074; 95% CI, 1.040‐4.137; *p* = 0.038) and stage III‐IV disease (HR, 4.263; 95% CI, 1.462‐12.431; *p* = 0.008) were significantly associated with increased risk of treatment failure, while development of grade ≥3 toxicity and cumulative cisplatin dose <200 mg/m^2^ were not associated with treatment failure. An age ≥70 years was associated with decreased risk of treatment failure (HR, 0.352; 95% CI, 0.143‐0.866; *p* = 0.023; Table 6).

**TABLE 6:** Univariate and multivariate analyses for risk of treatment failure

	Univariate			Multivariate		
	HR	95% CI	*p*‐value	HR	95% CI	*p*‐value
Age ≥70	0.625	0.277‐1.409	0.257	0.352	0.143‐0.866	0.023
PS 1‐2	1.713	0.925‐3.171	0.087	2.074	1.040‐4.137	0.038
Stage III‐IV	4.377	1.560‐12.284	0.005	4.263	1.462‐12.431	0.008
Grade ≥3 toxicity	1.123	0.612‐2.059	0.708	0.893	0.478‐1.670	0.724
Cumulative cisplatin dose <200 mg/m^2^	0.923	0.479‐1.777	0.810	1.235	0.600‐2.542	0.566

Abbreviations: CI, confidence internal; HR, hazard ratio; PS, Eastern Cooperative Oncology Group performance status.

## 4 DISCUSSION

This phase II study showed that CCRT that used individualized/dynamic cisplatin regimens based on patient age, PS, and patient/tumor responses during treatment resulted in favorable oncological outcomes with low toxicity in patients with HNSCC, even in those aged ≥70 years and with PS 1‐2.

In this study, the proportion of patients who received all planned cycles and ≥2 cycles was 79.3% and 97.3%, respectively, which represented similar or better compliance with chemotherapy regimens when compared with previous studies on cisplatin‐based CCRT that reported 59‐86% patients receiving all planned cycles and 79‐93% receiving ≥2 cycles.^1^, ^2^, ^3^, ^4^, ^11^, ^14^, ^16^ In addition, our individualized/dynamic cisplatin regimens had an incidence of grade 3‐4 toxicity of only 40.7% with no treatment‐related deaths, which was lower than the incidence of severe toxicity reported previous studies (77‐85%).^1^, ^3^, ^4^, ^16^ These results indicated that individualized/dynamic cisplatin regimens were highly tolerable and safe, further supporting their application in patients as a reasonable approach to minimizing treatment‐related complications of cisplatin‐based CCRT.

Even though individualized/dynamic cisplatin regimens present benefits in compliance and toxicity, a primary issue of these regimens is the possible decrease in the anticancer effect that can result from the decreased cumulative dose of cisplatin. Given that the current consensus of the target cumulative dose was 200 mg/m^2^, we intended to achieve at least 200 mg/m^2^ of a cumulative cisplatin dose for most patients if they received three cycles of chemotherapy, except in a minority of patients who were aged ≥80 years and with PS 2.^13^, ^19^, ^20^ As a result, the mean cumulative cisplatin dose was 212.3 mg/m^2^, suggesting that the study regimen can provide an adequate exposure to cisplatin in spite of the individualized and dynamic dose reduction during treatment. However, for some patients aged ≥80 years or with PS 2, we aimed for 180 mg/m^2^ as a target cumulative cisplatin dose to balance possible cisplatin‐related harms and benefits. Indeed, controversy still exists concerning whether the improved survival of patients receiving ≥200 mg/m^2^ was truly due to the dose‐dependent effectiveness of cisplatin or merely attributable to favorable patient characteristics, such as younger age or better PS, that enabled the administration of ≥200 mg/m^2^ cisplatin.^13^, ^19^ In the present study, Cox regression analysis showed that receiving <200 mg/m^2^ cisplatin was not associated with treatment failure, while eighth AJCC stage III‐IV disease and PS 1‐2 were demonstrated as independent risk factors of treatment failure. In fact, our indication for dose reduction‐included positive tumor response during CCRT, and 25 patients (16.7%) with excellent tumor response received reduced doses of cisplatin accordingly. Therefore, given the individuality and dynamics of the study protocol, the correlation between the cumulative cisplatin dose and oncological outcomes could not be verified in the present study. However, it is important to note that among the 25 patients who received a reduced cisplatin dose based on their excellent tumor response during CCRT, no treatment failure occurred. Therefore, we believe that cisplatin dose modification based on tumor response during CCRT was a feasible and reliable approach to minimize toxicity without compromising anticancer effects by providing an effective dose of cisplatin that fit the individual patient.

The oncological outcomes for the 2‐year LR control, PFS, and OS were 81.7%, 73.0%, and 86.1%, respectively. Given that these outcomes have been reported in ranges of 58‐84%, 47‐69%, and 41‐73%, respectively, the major oncological outcomes of the present study were comparable or superior to those of previous studies.^2^, ^3^, ^4^, ^10^, ^14^, ^16^ In addition, because one of the main goals of chemotherapy is to control distant metastases, there was a concern that the reduced dose protocol used in the present study would be associated with an increased risk of distant failure. However, the 2‐year distant control rate was 89.2%, which was also comparable to rates of 73‐92% reported in previous studies.^2^, ^3^, ^4^, ^11^, ^16^ Therefore, all oncological results involving LR control, distant control, and survival outcomes suggest that the individualized/dynamic cisplatin regimens did not compromise any oncological benefits of the currently used CCRT regimens at the price of reducing toxicity.

In general, older patients and deteriorated PS are more vulnerable to treatment toxicity compared to younger patients with normal PS, indicating that age and PS level might lead to poor treatment compliance and survival outcomes.^6^, ^8^, ^18^, ^21^, ^22^ In the present study, however, patients aged ≥70 years and with PS 1‐2 achieved comparable treatment compliance and toxicities with those reported in patients aged <70 years and with PS 0. In patients aged ≥70 years, all oncological outcomes, including LR control, distant control, PFS, ultimate PFS, DSS, and OS, were comparable with those in patients aged <70 years, although the mean cumulative dose was significantly lower in patients aged ≥70 years. These results suggest that CCRT using individualized/dynamic cisplatin regimen can be used safely and effectively, even in patients aged ≥70 years who are considered to be at high risk for conventional high‐dose cisplatin regimens. However, in patients with PS 1‐2, all oncological outcomes, except distant control, were worse than those in patients with PS 0, indicating that any deteriorated PS is a major poor prognostic factor of CCRT in patients with HNSCC.^9^, ^23^, ^24^ Furthermore, the results from subgroup analyses implied that chronological age per se is neither an absolute contraindication for cisplatin‐based CCRT nor a true risk factor for negative oncological outcomes. However, PS that represents a patient's functional age or comorbidities is a more important factor for making decisions about cisplatin‐based CCRT and predicting their prognosis ^6^, ^24^, ^25^, ^26^

This study had several limitations. First, we reported SCCs of all head and neck sites, including the nasopharynx, oral cavity, and nasal cavity/paranasal sinus, when cisplatin‐based CCRT was indicated. Thus, a direct comparison of our oncological results with other studies that mainly involved the oropharynx, larynx, and/or hypopharynx would be difficult. Second, the number of enrolled patients was relatively small and did not include a control group; therefore, the results of the present study cannot be generalized. Despite these limitations, given that cisplatin‐based CCRT is a major treatment modality for SCCs of all head and neck sites, and the aim of this study was to evaluate the oncological feasibility of individualized/dynamic cisplatin regimens, rather than demonstrate its superiority over conventional regimens, the protocols and results of this phase II study represent a good basis for establishing a standard protocol for cisplatin dose reduction and designing future phase III randomized controlled trials.^5^, ^7^

In conclusion, we found that CCRT using individualized/dynamic cisplatin regimens based on patient age, PS, and patient/tumor responses during treatment was oncologically safe and effective in patients with HNSCC, even in patients who were ≥70 years of age and had PS 1‐2. Large randomized controlled trials are necessary to confirm the results of the present study.

## AUTHOR CONTRIBUTIONS

Dongbin Ahn: Conceptualization, methodology, patient enrollment, data curation, visualization, statistical analyses, writing‐original draft, writing‐review, editing, data curation, and visualization. Gil Joon Lee: Patient enrollment, investigation, and resources. Jin Ho Sohn: Patient enrollment and resources. Jeong Eun Lee: Patient enrollment and supervision.

## Data Availability

The data that support the findings of this study are available from the corresponding author upon reasonable request.
